# Tafazzin (TAZ) promotes the tumorigenicity of cervical cancer cells and inhibits apoptosis

**DOI:** 10.1371/journal.pone.0177171

**Published:** 2017-05-10

**Authors:** Mei Chen, Yuan Zhang, Peng-Sheng Zheng

**Affiliations:** 1 The Department of Reproductive Medicine, The First Affiliated Hospital of Medical College, Xi’an Jiaotong University, Xi’an, China; 2 The Section of Cancer Research, Key Laboratory of Environment and Genes Related to Diseases, Ministry of Education of the People’s Republic of China, Xi’an, China; Institute of Biochemistry and Biotechnology, TAIWAN

## Abstract

Tafazzin (TAZ) is often aberrantly expressed in some cancers, including rectal cancer and thyroid neoplasms. However, the function of TAZ in cervical cancer cells remains unknown. This study aims to explore the expression and function of TAZ in cervical cancer cells. Here, we determined the expression of TAZ protein in normal cervical tissue (NC, n = 27), high-grade squamous intraepithelial lesions (HSIL, n = 26) and squamous cervical carcinoma (SCC, n = 41) by immunohistochemistry, the expression of TAZ protein gradually increased from NC to HSIL to SCC. TAZ was overexpressed or down-regulated in cervical cancer cells by stably transfecting a TAZ-expressing plasmid or a shRNA plasmid targeting TAZ. *In vitro*, the cell growth curves and MTT assays showed that TAZ may promote the growth and viability of cervical cancer cells. *In vivo*, xenografts experiment showed that TAZ may increase tumor-forming ability. The percentage of apoptosis cells analyzed by FACS and TUNEL assays consistently showed that TAZ inhibits apoptosis in cervical cancer cells. Furthermore, the Cleaved Caspase 9 and Cleaved Caspase 3 were down-regulated by TAZ in cervical cancer cells. Taken together, this study demonstrated that TAZ is overexpressed in cervical cancer and may promote tumorigenicity of cervical cancer cells and inhibit apoptosis.

## Introduction

Cervical cancer is a common cancer that contributes to cancer-related death in females[1]. Despite advances in detection and prevention, cervical cancer remains the third most frequently diagnosed female cancer worldwide. Approximately 80% of cervical cancer cases occur in developing countries, where approximately 270,000 women die from cervical cancer annually[2, 3]. Although infection with high-risk human papillomaviruses (HPV) is intimately related to the development of cervical cancer, HPV is not sufficient for cervical carcinogenesis and tumor progression[4–6]. Researchers have reported that the aggressive and proliferative nature of human cervical cancer is related to various factors that activate oncogenes and inactivate tumor suppressor genes[7–12].

The *tafazzin (TAZ)* gene is located on the distal region of chromosome Xq28 and encodes the tafazzin protein, which has an amino acid sequence homologous to acyltransferases[13]. TAZ is a mitochondrial protein localized in the mitochondrial membrane and plays a critical role in the remodeling of cardiolipin, a major lipid in the mitochondrial membrane[14]. Studies have shown that TAZ mutations can cause Barth syndrome, a rare and fatal X-linked genetic disorder[15]. In recent years, overexpression of TAZ has been observed in several tumors, including colon cancer[16], rectal cancer[17] and thyroid neoplasms[18]. Additionally, abnormal TAZ expression combined with higher IL-6 expression was found to promote inflammatory responses, which are commonly considered a predisposition factor for cancer progression[19]. However, the function of TAZ in cervical carcinogenesis is still not fully understood. Here, we explored the function and mechanism of TAZ in cervical cancer.

In the present study, TAZ protein expression was found to gradually increase in the progression of cervical carcinoma, as detected by IHC and Western blot. Furthermore, TAZ was verified to be able to promote cell growth both in vitro and in vivo and inhibit apoptosis in cervical cancer cells, providing preliminary evidence that TAZ contributes to cervical carcinogenesis.

## Materials and methods

### Human tissue samples and ethics statement

A total of 27 normal cervical samples (NC), 26 high-grade squamous intraepithelial lesions (HSIL) and 41 squamous cervical cancer samples (SCC) were obtained from patients at the First Affiliated Hospital of Xi’an Jiaotong University Medical College from 2008 to 2014. No subject had received chemotherapy, immunotherapy or radiotherapy before specimen collection. Histological classifications and clinical staging were based on the International Federation of Gynecology and Obstetrics classification system. The study was approved by the Ethics Committee of the Medical College of Xi’an Jiaotong University, and written informed consent was obtained from all subjects before sample collection.

### Cell lines and cell culture

Human cervical cancer cell lines (HeLa, SiHa, C33A, CaSki, HT-3) were purchased from the American Type Culture Collection (ATCC, Rockville, MD, USA) in 2007 and cultured at 37°C with 5% CO_2_ in our lab. The HeLa, SiHa and C33A cells were cultured in Dulbecco’s Modified Eagle’s Medium (DMEM, Sigma- Aldrich, USA. CaSki cells were cultured in RPMI1640 (Sigma-Aldrich, USA). HT-3 cells were cultured in McCoy’s 5A (Sigma-Aldrich, USA). All media was supplemented with 10% heat-inactivated fetal bovine serum (FBS, Invitrogen, Carlsbad, CA, USA).

### Immunostaining

Using a routine immunohistochemistry protocol, the specimens were fixed in 10% buffered formalin and embedded in paraffin. Then, 4 μm sections of the tissue samples were deparaffinized in xylene and rehydrated through descending concentrations of ethanol. Antigen retrieval was performed by heating in 10 mM citrate buffer (pH 6.0) for 2 minutes. The sections were then treated with 3% hydrogen peroxide to block endogenous peroxidases. After washing with phosphate-buffered saline (PBS) at room temperature, the sections were incubated overnight at 4°C with a rabbit polyclonal antibody against human TAZ (1:100 dilution; ab93362; Epitomics, USA). The sections were incubated with horseradish peroxidase-conjugated secondary antibody for 30 minutes at room temperature, followed by 3,3’-diaminobenzidine development. After that, the sections were counterstained with hematoxylin. As a negative control, the primary antibody was replaced with PBS. All slides were examined under an Olympus-CX31 microscope (Olympus, Tokyo, Japan) by two separate researchers. The staining intensity was scored as follows: 0 (negative), 1 (weak), 2 (moderate), 3 (strong). According to the percentage of positively stained cells, the staining extent was scored as 0 (0%), 1 (1%–25%), 2 (26%–50%), 3 (51%–75%) and 4 (76%–100%). The final immunoreactivity score (IRS) equaled the intensity score multiplied by the quantity score. The staining of TAZ was stratified into two categories according to the IRS: negative (0–4) and positive (5–12).

For the immunocytochemistry experiments, cells were seeded on autoclaved cover slips and, after 48 hours, fixed with 4% paraformaldehyde for 30 minutes and then permeabilized with 0.2% Triton X-100 for 20 minutes at room temperature. The staining procedure was similar to the immunohistochemistry process described above.

### Western blotting

Lysates from cells and fresh tissue were prepared on ice using lysis buffer (150 mM NaCl; 50 mM Tris-HCl, pH 7.4; 2 mM EDTA; 1% NP-40; and 0.1% SDS) containing a protease inhibitor cocktail (Complete Mini; Roche Diagnostics, Branchburg, NJ, USA). A BCA Protein Assay Reagent (Pierce, Rockford, IL, USA) was used to determine the protein concentrations. A total of 50 μg of protein samples was separated using SDS-PAGE, then transferred to PVDF membranes (Millipore, Billerica, MA, USA). The membranes were then blocked in 5% fat-free milk for one hour. The appropriate primary antibodies were incubated with the membranes at 4°C overnight. The primary antibodies included TAZ (1:500 dilution; ab93362; Epitomics, USA), Cleaved Caspase 9 (1:800 dilution; sc-56076, Santa Cruz, USA), Cleaved Caspase 3 (1:1000 dilution; #9661, Cell Signaling Technology, USA) and GAPDH (1:1000 dilution; sc-47724, Santa Cruz, USA). This was followed by secondary incubation using a secondary antibody, either horseradish peroxidase-conjugated anti-rabbit or anti-mouse IgG (Thermo Fisher Scientific, New York, NY, USA). The proteins were visualized on X-ray film with an enhanced chemiluminescence reagent (Millipore, Billerica, MA, USA). The relative densities of the Western blot bands were measured using the Alpha View system (Cell Biosciences, Santa Clara, CA, USA). The Western blot results were normalized to those of GAPDH for quantification purposes.

### Vector construction and transfection

The coding sequence (CDS) of the human TAZ gene was amplified from the cDNA of cervical cancer cell line SiHa by polymerase chain reaction (PCR) using the following primers:

F 5′-GGAAGATCTACCATGCCTCTGCACGTGAAGTGGC-3′;

R 5′-GTTGTCGACCTATCTCCCAGGCTGGAGGTGGTTG-3’.

The TAZ CDS fragment was subsequently cloned into the pCAG-PIRES2-AcGFP1 expression vector (Clontech, Mountain View, CA, USA) at the sites of *SalI and BglII* (TaKaRa, Tokyo, Japan). To construct a small interfering RNA expression vector that expressed TAZ-specific short hairpin RNA (shRNA), the following three TAZ sequences were used, which were obtained from GenePharma Co., Ltd. (Shanghai, China):

TAZ-Homo-897: 5′-GCCTGATTGCTGAGTGTCATC-3′;

TAZ-Homo-943: 5′-GCATGTCGGAATGAATGACGT-3′;

TAZ-Homo-1105: 5′-GGACTTCATTCAAGAGGAATT-3′.

Following the manufacturer’s instructions, the TAZ overexpression vectors were transfected into SiHa cells and the TAZ shRNA vectors were transfected into SiHa and HeLa cells using the Lipofectamine 2000 reagent (Invitrogen, Carlsbad, CA, USA). The drug-resistant colonies were collected, expanded and identified after the transfected cells were treated for 3 weeks with G418 (Calbiochem, La Jolla, CA, USA).

### Cell growth and cell viability assays

Cervical cancer cells were seeded in 6-well plates at a concentration of 1×10^4^ cells/well and cultured for 7 days. After harvesting on days 1, 3, 5 and 7 days the number of cells was counted using a hemocytometer under light microscopy, and cell growth curves were charted to assess cell growth. To assess cell viability, 3-(4,5-dimethylthiazole-yl)-2,5-diphenyl tetrazolium bromide (MTT, Sigma-Aldrich) was used. Cells were seeded at a density of 1000 cells per well in 96-well plates and cultured for 7 days. Six parallel samples were used for each condition. Assays were performed according to the manufacturer’s protocol.

### Flow cytometry analysis

FACS Calibur flow cytometry (Becton Dickinson, Franklin Lakes, NJ, USA) was used to analyze the cell cycle. In brief, approximately 1×10^6^ cells were collected and fixed in cool 70% ethanol at 4°C overnight. After washing twice with PBS, the cells were incubated in 1 mL of staining solution including 20 mg/mL propidium iodide (Sigma-Aldrich, St. Louis, MO, USA) and 10 U/mL RNase A for 30 minutes at room temperature. Then, cell cycle progression was analyzed by FACS.

For apoptosis analysis, a PE Annexin V Apoptosis Detection Kit (559763, BD, Franklin Lakes, NJ, USA) was used for flow cytometry. After being cultured for 48 hours, the cells were harvested and washed with PBS, then incubated with PE Annexin V and 7-AAD for 10 minutes in the dark. Finally, FACS (Becton Dickinson, Franklin Lakes, NJ, USA) was used to detect the stained cells.

### Tumor xenograft experiment

Female BALB/c-nude mice that were 4 to 6 weeks old were obtained from the Model Animal Research Center of Nanjing University (Nanjing, China) and housed in a SFP room. A total of 1×10^6^ tumor cells were injected into the subcutis on the dorsum of each mouse. The tumor sizes were measured every three days using vernier calipers, and tumor volumes were calculated with the standard formula: length×width^2^/2. At the end of the experiment, the mice were killed by cervical vertebra dislocation and the tumors were weighed. Then, the tissue from the xenograft tumors were fixed and paraffin-embedded for histological analysis. All animal studies were evaluated and approved by the Animal Care and Use Committee of the Medical School of Xi’an Jiaotong University.

### TUNEL assay

Apoptosis was detected by using the In Situ Cell Death Detection Kit, POD (Roche Corporation, Switzerland) according to the manufacturer’s instructions. In brief, the paraffin-embedded tissue was incubated with proteinase K at 37°C for 15 minutes after dewaxing and rehydrating and then incubated with the TUNEL reaction mixture (50 μl Enzyme Solution mixed with 450 μl Label Solution) for 60 minutes at 37°C with a humidified atmosphere in the dark. After being washed 3 times with PBS for 5 minutes each, converter-POD was added and the slides were incubated in a humidified chamber for 30 minutes at 37°C. Finally, the samples were incubated with DAB substrate for 10 minutes at room temperature. The total number of cells with TUNEL-positive nuclei was counted under a light microscope.

### Statistical analysis

Statistical analysis was performed using SPSS software version 16.0 (SPSS Inc., Chicago, IL, USA). All data were expressed as the mean ± standard deviation of the mean (SD). For group comparisons, Student’s t-test, Mann-Whitney U test, one-way ANOVA and chi-square tests were performed. *P*<0.05 was defined as statistically significant.

## Results

### The expression of TAZ in human normal cervix and various cervical lesions

**Fig 1:**
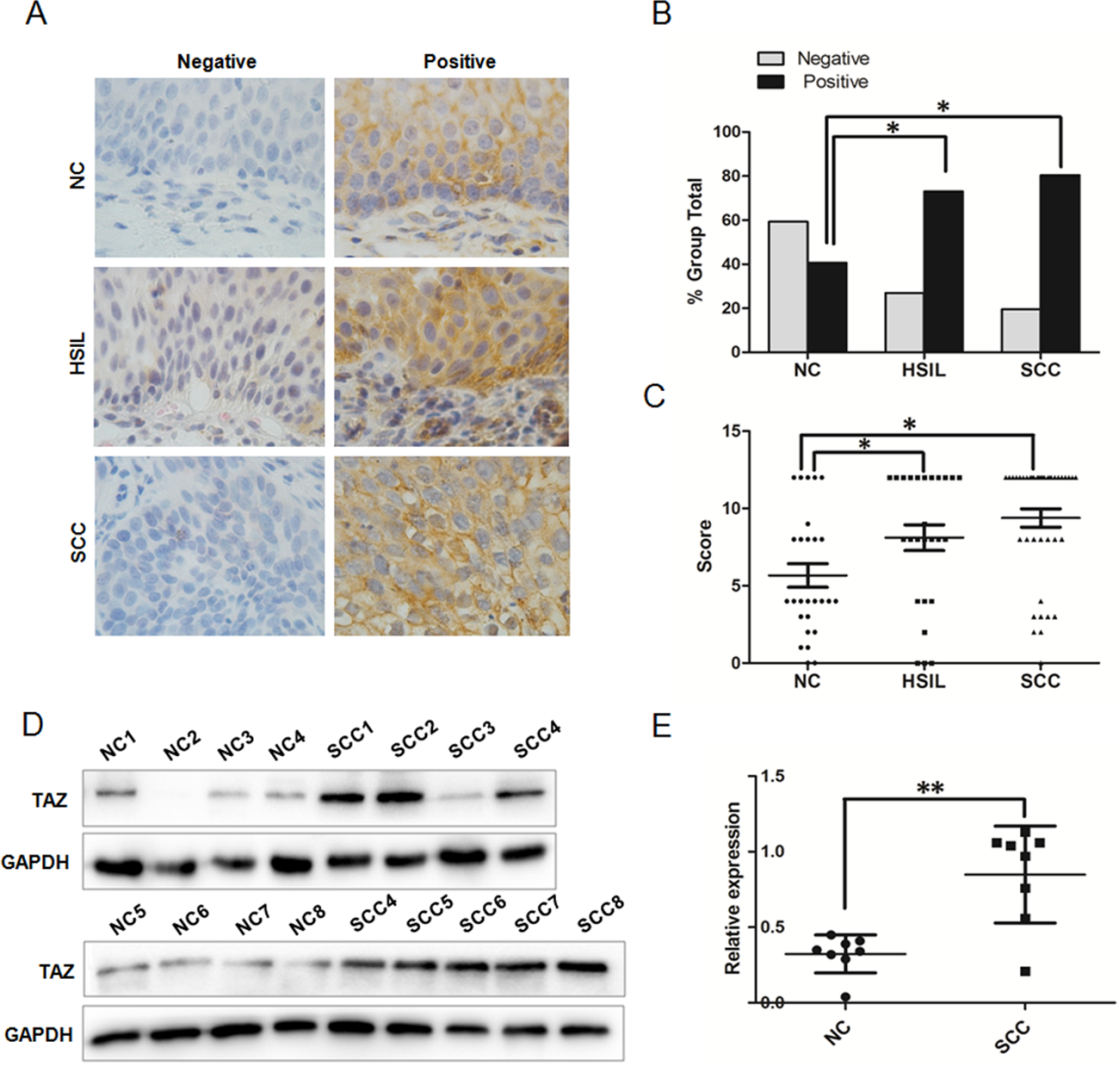
TAZ expression in human normal cervical tissue and in various cervical lesions. **(A)** Immunohistochemical (IHC) staining showing TAZ expression in normal cervix, high-grade squamous intraepithelial lesion and squamous cervical cancer (magnification, ×100). **(B)** TAZ staining is classified into 2 categories (negative and positive). Bar graph showing TAZ-positive percentage and TAZ-negative percentage of 27 normal cervix samples, 26 high-grade squamous intraepithelial lesions and 41 squamous cervical cancer lesions. **(C)** The IHC score of TAZ staining in normal cervix, high-grade squamous intraepithelial lesions and squamous cervical cancer. **(D)** Western blot analysis of TAZ expression in 8 fresh normal cervix samples and 8 squamous cervical cancer samples. representative blots are shown. **(E)** The relative expression of TAZ in normal cervix samples and squamous cervical cancer samples. GAPDH was used as a loading control. NC: normal cervix; HSIL: high-grade squamous intraepithelial lesion; SCC: squamous cervical cancer. Values are shown as the mean±SD, * *P*<0.05 *vs*. control; ** *P*<0.01 *vs*. control.

To understand the endogenous TAZ expression in cervical carcinogenesis, we detected the expression of TAZ in 27 NC, 26 HSIL and 41 SCC samples by immunohistochemistry. As shown in Fig 1A, TAZ staining was observed in the cytoplasm of positive cells, both in the NC tissue and various cervical lesions. According to the established method of IRS, the rate of positive TAZ expression was 40.74% (11 of 27) in the NC samples and was significantly increased to 73.08% (19 of 26) in HSIL lesions and 80.49% (33 of 41) in SCC lesions (Table 1 and Fig 1B, *P*<0.05). Additionally, the IHC score of the TAZ staining gradually increased from 5.67±3.97 in NC tissue to 8.11±4.26 in HSIL lesions and 9.30±3.79 in SCC lesions (Fig 1C and S1 Table, *P*<0.05). The expression of TAZ protein in 8 fresh NC samples and 8 fresh SCC lesions from patients undergoing surgery was detected by Western blot (Fig 1D), and the level of TAZ expression related to GAPDH was higher in SCC than that in normal cervical tissue (Fig 1E, *P*<0.01). All these results indicate that TAZ protein is up-regulated in SCC, suggesting that the TAZ may promote cervical carcinogenesis.

**Table 1:** TAZ expression in different cervical tissue specimens.

Specimens	Total	TAZ Staining		*P-*value
		Negative, No. (%)	Strong, No. (%)	
NC	27	16(59.26)	11(40.74)	
HSIL	26	7(26.92)	19(73.08)	<0.05^a^
SCC	41	8(19.51)	33(80.49)	<0.05^b^

NC: normal cervix; HSIL: high-grade squamous intraepithelial lesion; SCC: squamous cervical cancer. a: HSIL versus NC; b: SCC versus NC.

### TAZ promotes the growth of cervical cancer cell in vitro

**Fig 2:**
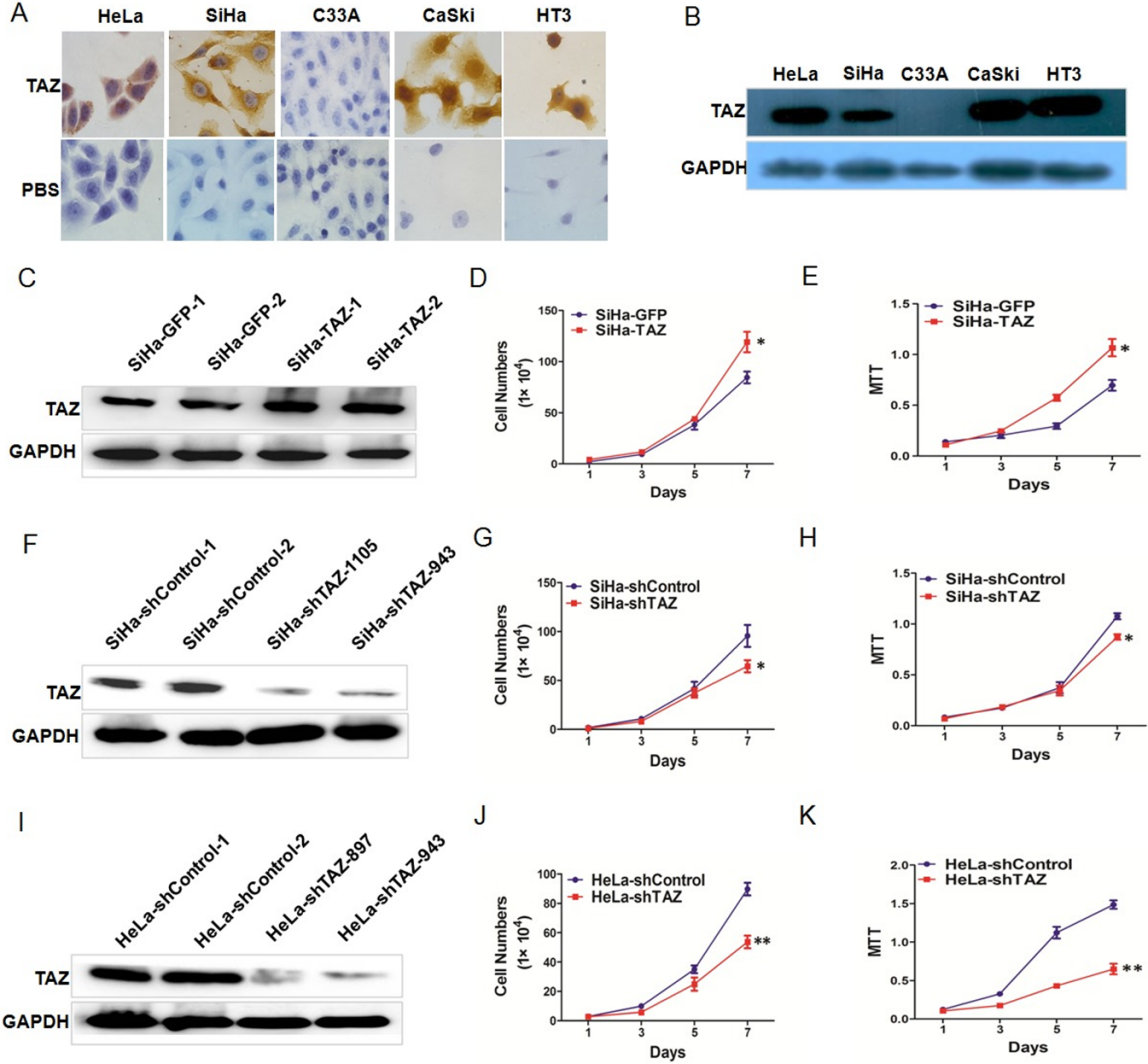
TAZ increases the growth of cervical cancer cells *in vitro*. **(A)** Immunocytochemical staining showing TAZ expression in HeLa, SiHa, C33A, Caski and HT3 cells (magnification, ×100). (**B)** Western blot analysis of TAZ expression in HeLa, SiHa, C33A, Caski and HT3 cells. Stably transfected cell lines were identified by western blotting: **(C)** control (SiHa-GFP) and TAZ-overexpressing SiHa (SiHa-TAZ) cells; (F) control(SiHa-shControl) and TAZ-knockdown SiHa (SiHa-shTAZ) cells; (I) control(HeLa-shControl) and TAZ-knockdown HeLa (HeLa -shTAZ) cells. The growth and viability of cells were detected using growth curves and 3-(4,5-dimethylthiazole-yl)-2,5-diphenyl tetrazolium bromide (MTT) assay in SiHa-GFP and SiHa -TAZ cells **(D, E)**, SiHa-shControl and SiHa-shTAZ cells **(G, H)**, and HeLa-shControl and HeLa-shTAZ cells **(J, K)**. Values are shown as the mean±SD obtained from 3 separated experiments, * *P*<0.05 *vs*. control; ** *P*<0.01 *vs*. control.

What is the function of TAZ in cervical cancer cells? The expression of TAZ was observed by immunocytochemistry and Western blotting in the cervical cancer cell lines SiHa, HeLa, C33A, CaSki and HT-3 (Fig 2A and 2B). TAZ was up-regulated in SiHa cells by stable transfection with a TAZ-expressing plasmid (Fig 2C). TAZ was silenced in SiHa and HeLa cells by transfection with an shRNA plasmid targeting TAZ (Fig 2F and 2I). To uncover the potential effect of TAZ on the growth and viability of cervical cancer cells, cell growth curves and MTT assays were performed *in vitro* to evaluate cell growth and viability. The cell growth curve assay and the MTT assay showed that TAZ-overexpressing SiHa (SiHa-TAZ) cells had significantly stronger cell growth and cell viability than the control cells (SiHa-GFP) (Fig 2D and 2E, *P*<0.05). However, the TAZ-silenced SiHa (SiHa-shTAZ) and HeLa (HeLa-shTAZ) cells had significantly weaker cell growth and cell viability than the control (SiHa-shControl and HeLa-shControl) cells did (Fig 2G, 2H, 2J and 2K, *P*<0.05). The above results demonstrate that TAZ may promote the growth of cervical cancer cells in vitro.

### TAZ enhances the tumor formation of cervical cancer cells *in vivo*

**Fig 3:**
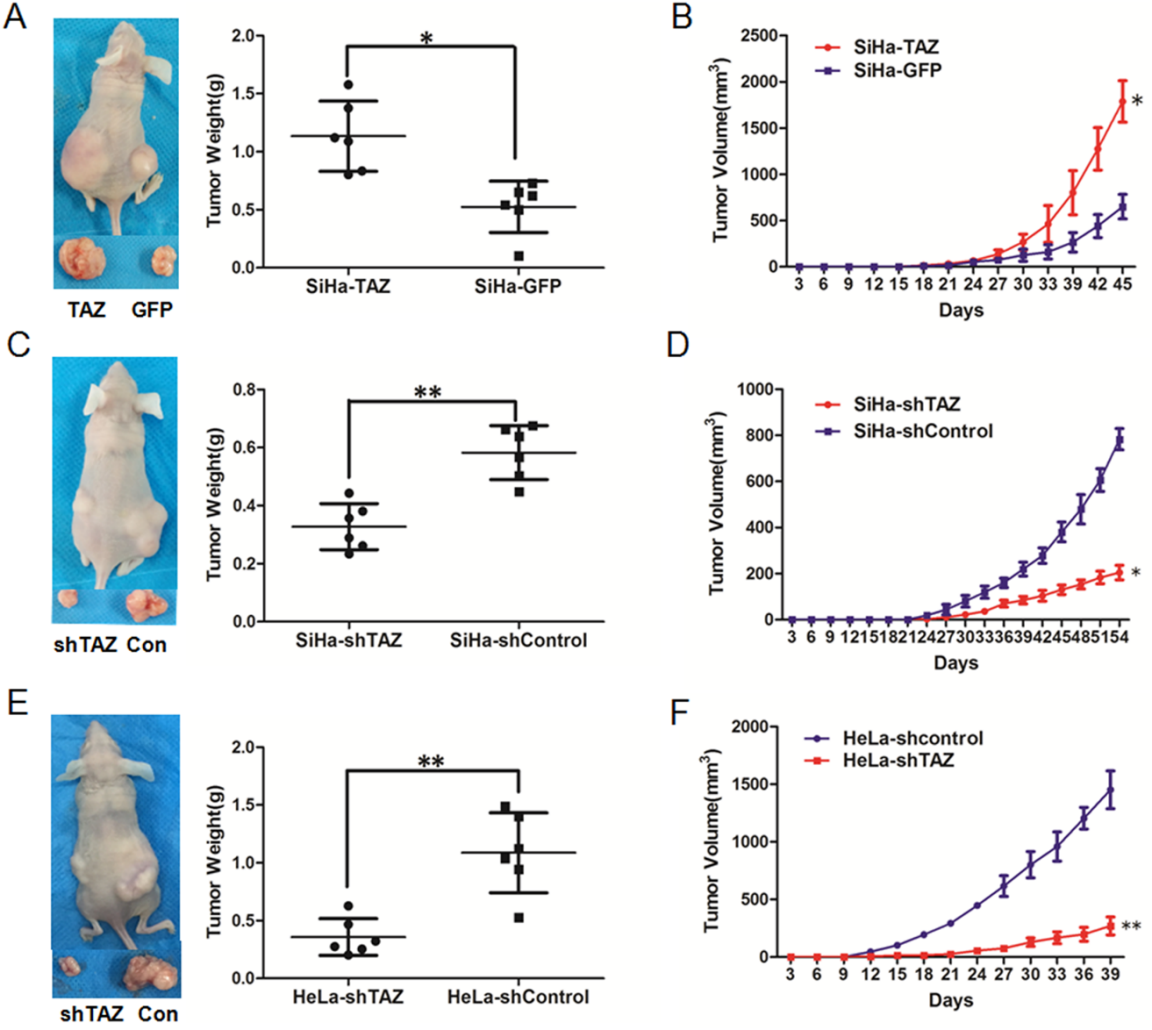
TAZ enhances the growth of cervical cancer xenografts in vivo. Tumor formation assays were performed with 6 mice per group. The tumor growth curves were calculated after injection into female nude mice based on monitoring performed every three days:(B) SiHa-GFP and SiHa-TAZ cells; (D) SiHa-shControl and SiHa-shTAZ cells; (F) HeLa-shControl and HeLa-shTAZ cells. The xenograft tumors were dissociated and weighed at the end of experiment: (A) SiHa-GFP and SiHa-TAZ cells; (C) SiHa-shControl and SiHa-shTAZ cells; (E) HeLa-shControl and HeLa-shTAZ cells. Values are shown as the mean±SD,* *P*<0.05 *vs*. control; ** *P*<0.01 *vs*. control.

Next, we focused on the effects of TAZ on tumor formation of cervical cancer cells by performing a xenograft experiment in female BALB/c nude mice. A total of 1×10^6^ TAZ-modified HeLa and SiHa cells and their respective control cells were injected into the subcutis on the dorsum of each mouse at the same time. Tumor volume was then continuously measured every three days. The growth of the tumors formed by SiHa-TAZ cells was much faster than those formed by SiHa-GFP cells (Fig 3B, *P*<0.05). Furthermore, at the termination of the experiment, the average tumor weight was 1.13±0.30 g in the SiHa-TAZ cell group, which was much heavier than that in the SiHa-GFP cell group (0.52±0.22 g) (Fig 3A, *P*<0.05). The consistent effect of TAZ on the tumor formation of cervical cancer cells was found in TAZ-silenced SiHa and HeLa cells. The tumors formed by SiHa-shTAZ cells grew more slowly than the SiHa-shControl cells did (Fig 3D, *P*<0.05). Additionally, the average weight of tumors formed by SiHa-shTAZ cells (0.33±0.08 g) was lighter than the average weight of SiHa-shControl tumors (0.58±0.09 g, Fig 3C, *P*<0.05). Similarly, the silencing of TAZ in HeLa cells significantly inhibited tumor growth ability *in vivo* (Fig 3F, *P*<0.01); the average tumor weight was 0.36±0.16 g in the HeLa-shTAZ group and 1.09±0.35 g in the HeLa-shControl group (Fig 3E, *P*<0.01). These results suggest that TAZ may enhance cervical cancer tumor formation in vivo.

### TAZ inhibits cell apoptosis of cervical cancer cells

**Fig 4:**
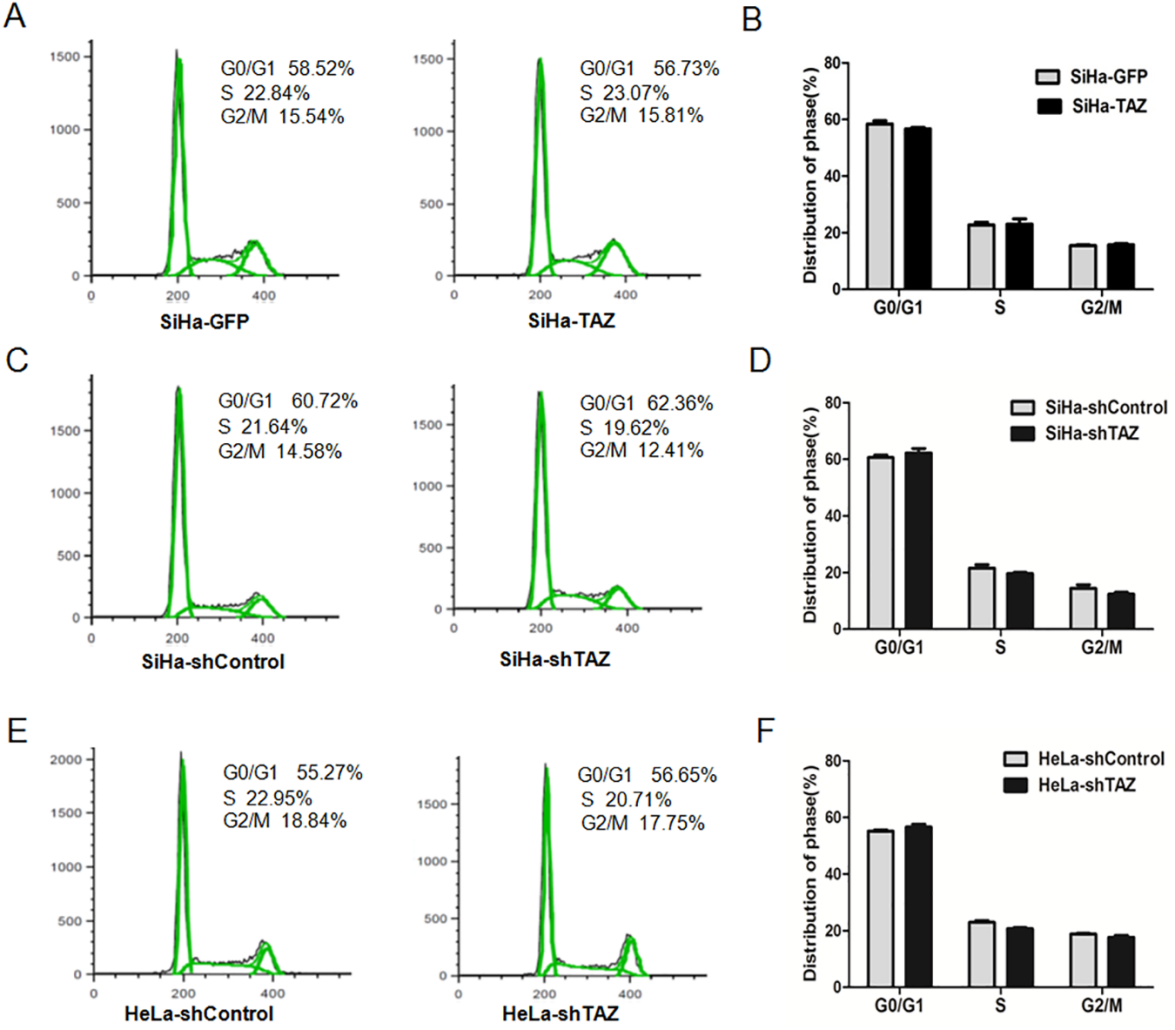
TAZ does not change the cell cycle distributions in TAZ-mediated cervical cancer cells. The cell cycle in TAZ-mediated cervical cancer cells were monitored with fluorescence-activated cell sorting (FACS) analysis **(A, C, E,)**. A quantitative analysis of the cell cycle distribution is shown. There was no significant difference in the G0/G1, S or G2/M phases between SiHa-GFP and SiHa -TAZ cells **(B)**, SiHa-shControl and SiHa-shTAZ cells **(D)**, and HeLa-shControl and HeLa-shTAZ cells **(F)**. Values are expressed as the mean±SD of three experiments in duplicate. * *P*<0.05 *vs*. control; ** *P*<0.01 *vs*. control.

To uncover the function of TAZ in cervical cancer cell growth, cell cycle distribution was monitored by FACS technology. As shown in Fig 4A and 4B, 56.73% of SiHa-TAZ cells were in the G0/G1 phase, 23.07% were in the S phase, and 15.81% were in the G2/M phase, which was not statistically different compared to the SiHa-GFP cells (58.52% in G0/G1, 22.84% in S, 15.54% in G2/M). Consistently, there was no significant difference in the cell cycle stages of the TAZ-silenced SiHa cells (62.36% in G0/G1, 19.62% in S, 12.41% in G2/M) and the SiHa-shControl cells (60.72% in G0/G1, 21.64% in S, 14.58% in G2/M; Fig 4C and 4D). Similarly, the cell cycle distribution of HeLa-shTAZ cells (56.65% in G0/G1, 20.71% in S, 17.75% in G2/M) was not significantly different from that of the HeLa-shControl cells (55.27% in G0/G1, 22.95% in S, 18.84% in G2/M; Fig 4E and 4F). These results suggest that TAZ is not involved in the cell cycle distribution of cervical cancer cells.

**Fig 5:**
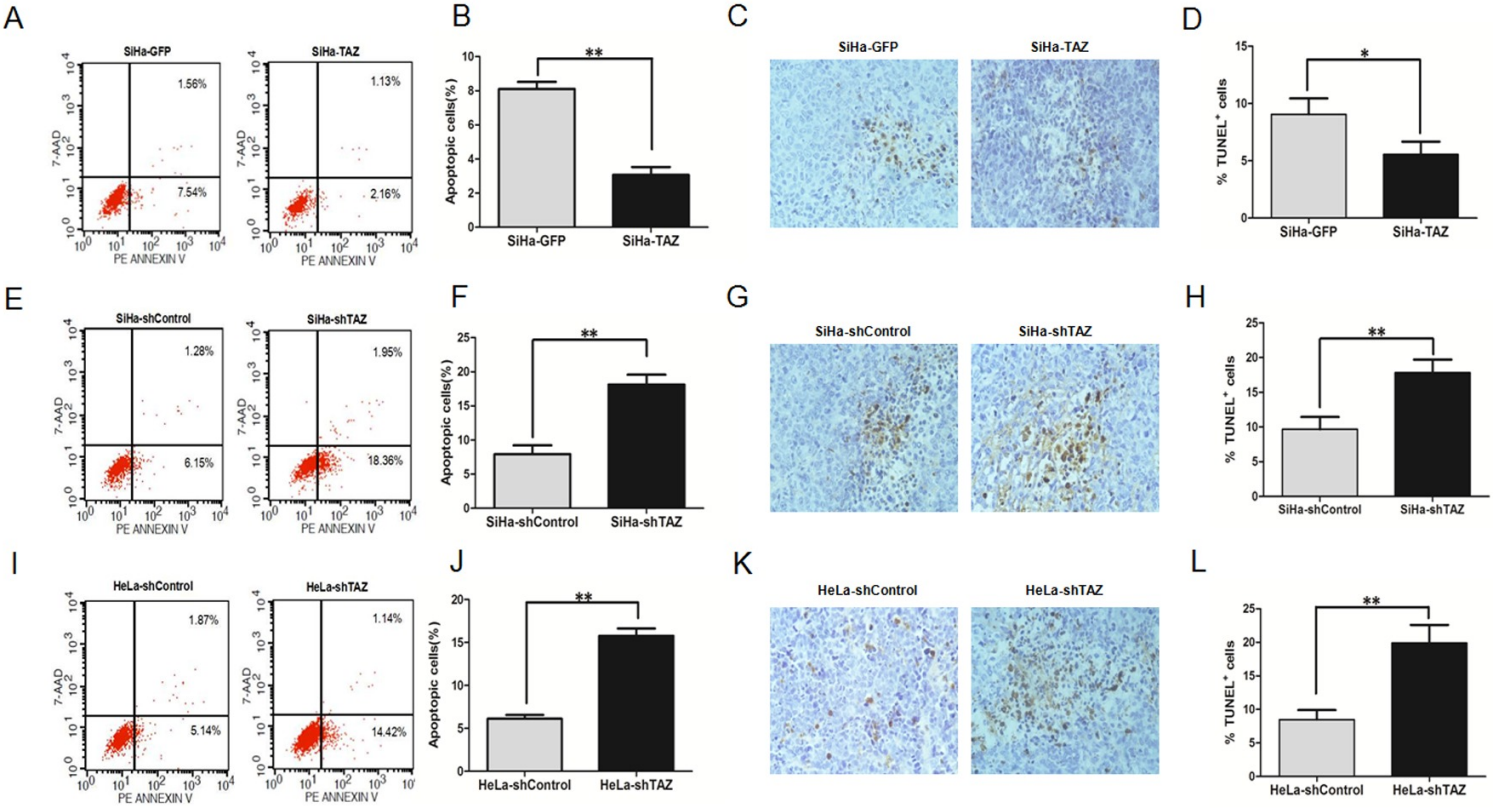
TAZ inhibits apoptosis in cervical cancer cells both in vitro and in vivo. Apoptosis of TAZ-mediated cervical cancer cells was monitored with fluorescence-activated cell sorting (FACS) analysis. The representative cell apoptosis histograms and the percentage of apoptosis cells of TAZ-mediated cervical cancer cells are shown: (A, B) SiHa-GFP and SiHa-TAZ cells; (E, F) SiHa-shControl and SiHa-shTAZ cells; (I, J) HeLa-shControl and HeLa-shTAZ cells. Values are expressed as the mean±SD of three experiments in duplicate. Apoptotic cell death in tumor xenografts formed by TAZ-mediated cervical cancer cells was measured by TUNEL assay, and representative micrographs are shown (magnification, ×40):(C) SiHa-GFP and SiHa-TAZ cells; (G) SiHa-shControl and SiHa-shTAZ cells; (K) HeLa-shControl and HeLa-shTAZ cells. Quantitative analysis of apoptosis cells in xenograft samples formed by TAZ-mediated cervical cancer cells: (D) SiHa-GFP and SiHa-TAZ cells; (H) SiHa-shControl and SiHa-shTAZ cells; (L) HeLa-shControl and HeLa-shTAZ cells. Six tumor samples were measured and analysed every group. Values are shown as the mean±SD. *P<0.05 *vs*. control; ** *P*<0.01 *vs*. control.

We then examined apoptosis in cervical cancer cells by FACS using the PE annexin V /7-AAD double staining kit in TAZ-modified cervical cancer cells and the corresponding control cells, respectively. We found that the percentage of SiHa-TAZ cells in apoptosis was 2.16%, which was lower than that of SiHa-GFP cells (7.54%, Fig 5A and 5B, *P*<0.01). Moreover, the percentage of apoptosis cells in TAZ-silenced SiHa cells was 18.36%, which was higher than that in SiHa-shControl cells(6.15%, Fig 5E and 5F, *P*<0.01), the percentage of apoptosis cells in TAZ-silenced HeLa cells was 14.42%, which was higher than that in HeLa-shControl cells(5.14%, Fig 5I and 5J, *P*<0.01). These results indicate that TAZ may inhibit cervical cancer cell apoptosis.

To further investigate whether TAZ inhibits cell apoptosis in the tumor formation of cervical cancer cells, a TUNEL assay was performed in xenograft tumor tissues formed by TAZ-mediated SiHa and HeLa cells. The percentage of TUNEL-positive cells in tumor tissues formed by SiHa-TAZ cells(5.55%) was significantly lower than that in tumor tissues formed by SiHa-GFP cells (9.05%, Fig 5C and 5D, *P*<0.05). The percentage of TUNEL-positive cells in tumor tissues derived from TAZ-silenced SiHa cells was 17.85%, which was higher than that in tumor tissues formed by SiHa-shControl cells (9.68%, Fig 5G and 5H, *P*<0.01). Similarly, The percentage of TUNEL-positive cells in tumor tissues derived from TAZ-silenced HeLa cells was 19.90%, which was higher than that in tumor tissues formed by HeLa-shControl cells (8.48%, Fig 5K and 5L, *P*<0.01). These results indicate that TAZ inhibits apoptosis in cervical cancer cells both in vitro and in vivo.

### TAZ limits the cleavages of Caspase 9 and Caspase 3 in cervical cancer cells

**Fig 6:**
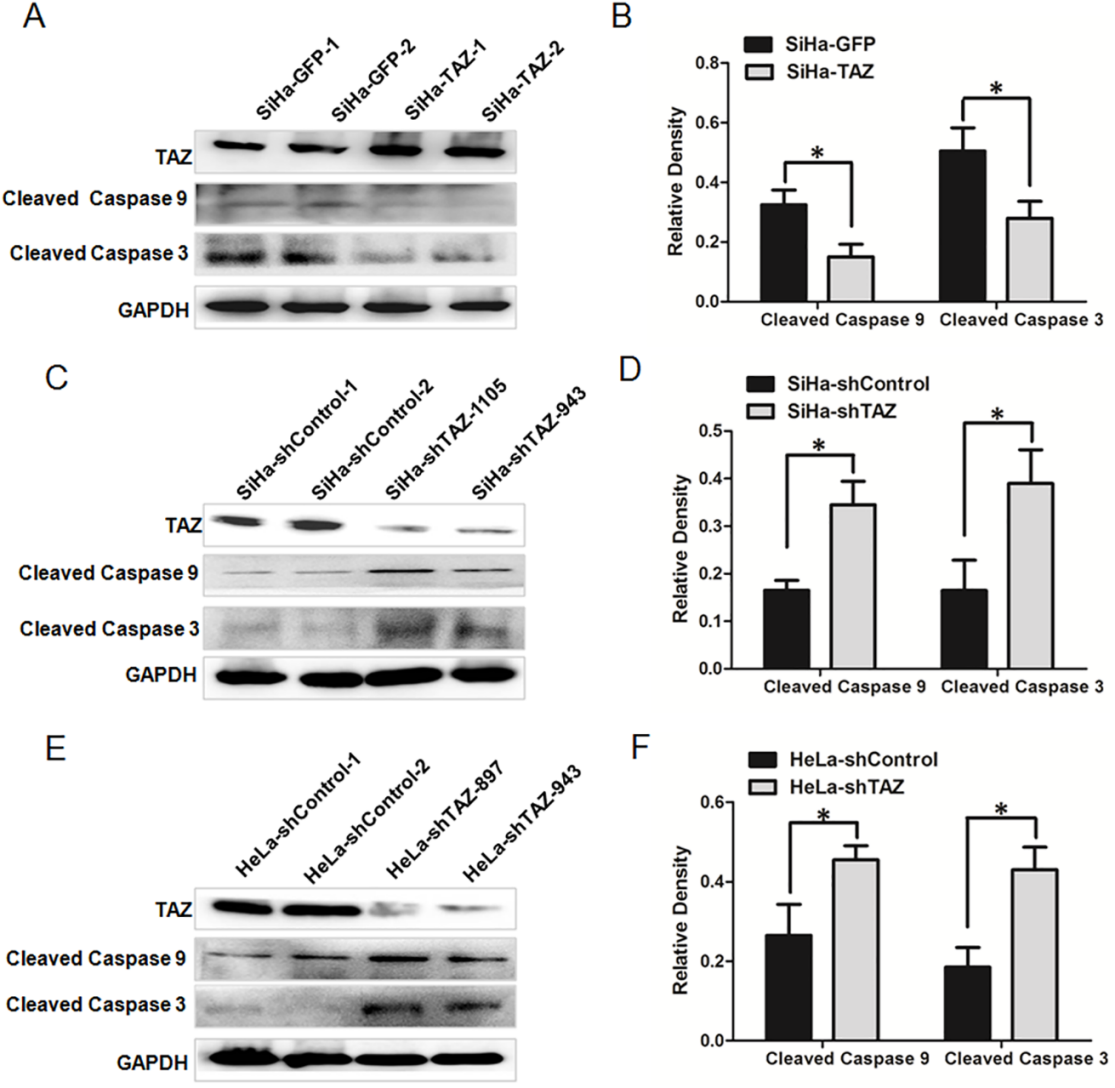
TAZ limits the cleavages of Caspase 9 and Caspase 3 in cervical cancer cells. Western blot analysis showing the expression of Cleaved Caspase 9 and Cleaved Caspase 3 in TAZ-mediated cervical cancer cells: **(A)** SiHa-GFP and SiHa-TAZ cells with the quantitative analysis **(B); (C) SiHa-**shControl and SiHa-shTAZ cells with the quantitative analysis **(D); (E)** HeLa-shControl and HeLa-shTAZ cells with the quantitative analysis **(F)**. Values are shown as the mean±SD of three experiments in duplicate. *P<0.05 *vs*. control; ** *P*<0.01 *vs*. control.

Caspase 9 and Caspase 3 have distinct roles in the intrinsic apoptotic pathways. First, Caspase 9 is activated by mitochondria-released cytochrome c, and then, Caspase 3 is activated[20]. To confirm the decreased induction of apoptosis in TAZ-expressing cervical cancer cells, Western blotting was performed to detect the expression of Cleaved Caspase 9 and Cleaved Caspase 3, which are involved in the mitochondrial apoptosis pathway. The relative expression of Cleaved Caspase 9 and Cleaved Caspase 3 in SiHa-TAZ cells was lower than that in SiHa-GFP cells (Fig 6A and 6B, *P*<0.05). In contrast, the expression of Cleaved Caspase 9 and Cleaved Caspase 3 in SiHa-shTAZ cells was higher than in SiHa-shControl cells (Fig 6C and 6D, *P*<0.05). Similarly, the expression of Cleaved Caspase 9 and Cleaved Caspase 3 was higher in HeLa-shTAZ cells than that in HeLa-shControl cells (Fig 6E and 6F, *P*<0.05). These results suggest that TAZ inhibits apoptosis via limited cleavages of Caspase 9 and Caspase 3 in cervical cancer cells.

## Discussion

TAZ, an important cardiolipin-remodeling enzyme, is involved in the maintenance of mitochondrial cardiolipin, which plays an important role in cellular metabolism, as exemplified by its involvement in mitochondrial energy production and apoptosis[14]. In recent years, TAZ has been observed to be overexpressed in several tumors, including thyroid carcinoma[18], rectal cancer[17], and colon cancer[16], suggesting it may function as a promoter in tumorigenesis. However, to date, the expression and mechanism of the involvement of TAZ in cervical cancer have not been thoroughly investigated.

Here, we found that TAZ expression gradually increased from NC (40.74%) to HSIL (73.08%) to SCC (80.49%), suggesting that TAZ may function to promote the development and progression of cervical cancer (Fig 1). The overexpression of TAZ in SiHa cells could enhance cell growth *in vitro and in vivo*, and reduced expression of TAZ in SiHa and HeLa cells could inhibit cell growth *in vitro* and *in vivo* (Figs 2 and 3). It has been previously reported that strong expression of the TAZ protein is related to rectal cancer development and RT response[16]. Our results *in vitro* and *in vivo* also support the hypothesis that TAZ is a tumor enhancer in cervical cancer.

To uncover how TAZ affects cervical cancer cell growth, cell cycle and apoptosis experiments were performed, and the results show that TAZ may promote cervical cancer growth and inhibit cell apoptosis, but do not affect the cell cycle distribution in cervical cancer cells. In Barth syndrome, cardiolipin remodeling due to TAZ gene mutations can inhibit cell apoptosis[15], suggesting that abnormal TAZ expression is related to apoptosis. In rectal cancer, strong TAZ expression was positively correlated with expression of Livin [17], which is an apoptosis inhibitory protein and has been found to be involved in tumorigenesis and metastasis in epithelial tumors[21]. In cervical cancer, we propose that TAZ accelerates cell growth and inhibits cell apoptosis.

In mammalian cells, the induction of apoptosis occurs via two distinct pathways, the extrinsic pathway and the intrinsic pathway. The intrinsic apoptotic cascade involves the release of apoptogenic factors from the mitochondria into the cytosol[22, 23]. In the intrinsic pathway, Caspase 9 is first activated by mitochondria-released *cytochrome c* and then activates Caspase 3. Cardiolipin is required for *cytochrome c* release from mitochondria[23, 24]. Cardiolipin, a phospholipid of the mitochondrial membrane, participates in several mitochondria-dependent apoptotic steps[24]. The maturation of cardiolipin requires the transacylase TAZ[25]. To further explore whether TAZ could regulate the expression of mitochondria-derived Caspases, which are important factors in the apoptosis pathway, we detected the levels of Cleaved Caspase 9 and Cleaved Caspase 3 proteins in distinct types of cervical cancer cells via Western blot. The expression of Cleaved Caspase 9 and Cleaved Caspase 3 was down-regulated in TAZ-over-expressing SiHa cells, whereas the levels of Cleaved Caspase 9 and Cleaved Caspase 3 were up-regulated in TAZ-knock-down SiHa and HeLa cells (Fig 6). These results show that TAZ may decrease the cleavages of Caspase 9 and Caspase 3, which are important apoptosis-related factors in cervical cancer cells.

In summary, our study demonstrates that TAZ promotes tumorigenesis of cervical cancer cells and inhibits cell apoptosis. Based on our results and previous literature, we propose that TAZ may inhibit the apoptosis of cervical cancer cells through the mitochondrial pathway, as characterized by the decreased cleavages of Caspase9 and Caspase3. However, the precise mechanism of TAZ-limited apoptosis need to be elucidated by further research (i.e. how does TAZ limits the cleavages of Caspase9 and Caspase3?). Our results suggest that TAZ promotes cell growth and tumor formaion in cervical cancer cells and may be a potential therapeutic target in certain types of cervical cancer.

## Supporting information

S1 Table The IHC score of the TAZ staining in human normal cervical tissue, high-grade squamous intraepithelial lesion and squamous cervical cancer. (XLSX)
